# Increased Virulence of an Epidemic Strain of Vesicular Stomatitis Virus Is Associated With Interference of the Innate Response in Pigs

**DOI:** 10.3389/fmicb.2018.01891

**Published:** 2018-08-15

**Authors:** Lauro Velazquez-Salinas, Steven J. Pauszek, Carolina Stenfeldt, Emily S. O’Hearn, Juan M. Pacheco, Manuel V. Borca, Antonio Verdugo-Rodriguez, Jonathan Arzt, Luis L. Rodriguez

**Affiliations:** ^1^Foreign Animal Disease Research Unit, Plum Island Animal Disease Center, United States Department of Agriculture–Agricultural Research Service, Greenport, NY, United States; ^2^College of Veterinary Medicine and Animal Science, National Autonomous University of Mexico, Mexico City, Mexico; ^3^Plum Island Animal Disease Center Research Participation Program, Oak Ridge Institute for Science and Education, Oak Ridge, TN, United States; ^4^Department of Veterinary Population Medicine, University of Minnesota, St. Paul, MN, United States; ^5^Foreign Animal Disease Diagnostic Laboratory, Veterinary Services, Animal and Plant Health Inspection Service, United States Department of Agriculture, Plum Island, NY, United States

**Keywords:** vesicular stomatitis, parthenogenesis, virulence, interferon, immune response, IL-6, TNF, epidemics

## Abstract

Vesicular stomatitis virus (VSV) causes sporadic outbreaks of vesicular disease in the southwestern United States. The intrinsic characteristics of epidemic strains associated with these outbreaks are poorly understood. In this study, we report the distinctive genomic and biological characteristics of an epidemic (NJ0612NME6) strain of VSV compared with an endemic (NJ0806VCB) strain. Genomic comparisons between the two strains revealed a total of 111 nucleotide differences (23 non-synonymous) with potentially relevant replacements located in the P, G, and L proteins. When tested in experimentally infected pigs, a natural host of VSV, the epidemic strain caused higher fever and an increased number of vesicular lesions compared to pigs infected with the endemic strain. Pigs infected with the epidemic strain showed decreased systemic antiviral activity (type I – IFN), lower antibody levels, higher levels of interleukin 6, and lower levels of tumor necrosis factor during the acute phase of disease compared to pigs infected with the endemic strain. Furthermore, we document the existence of an RNAemia phase in pigs experimentally infected with VSV and explored the cause for the lack of recovery of infectious virus from blood. Finally, the epidemic strain was shown to be more efficient in down-regulating transcription of IRF-7 in primary porcine macrophages. Collectively, the data shows that the epidemic strain of VSV we tested has an enhanced ability to modulate the innate immune response of the vertebrate host. Further studies are needed to examine other epidemic strains and what contributions a phenotype of increased virulence might have on the transmission of VSV during epizootics.

## Introduction

Vesicular stomatitis (VS) is caused by the arthropod-borne vesicular stomatitis virus (VSV, family Rhabdoviridae, genus *Vesiculovirus*). The non-segmented RNA viral genome of VSV encodes five structural proteins: nucleocapsid (N), phosphoprotein (P), matrix (M), glycoprotein (G), and the large RNA-dependent RNA polymerase (L) (Wagner and Rose, 1996; Dietzgen, 2012) along with two non-structural proteins (C and C′) of undetermined function encoded from overlapping reading frames in the P gene (Spiropoulou and Nichol, 1993). VSV causes most of the cases of vesicular disease reported in livestock (Rodriguez, 2002; Mead et al., 2009). Because its clinical signs in cattle and pigs are indistinguishable from those of foot-and-mouth disease (FMD), VS reports result in costly animal quarantines and trade embargoes (Velazquez-Salinas et al., 2014). Unlike FMD, VS also occurs in horses, resulting in animal movement restrictions that cause disruption to trade shows (Bridges et al., 1997; Timoney, 2016).

Taxonomically, VSV is classified into two distinct serotypes: New Jersey (VSNJV) and Indiana (VSIV) (Rodriguez, 2002; Dietzgen, 2012). Both of these serotypes have been reported from as far south as Bolivia to as far north as the United States. VSV is considered endemic from southern Mexico throughout Central America to northern South America, where multiple genetic lineages co-circulate each year. VS occurs less frequently in Mexico’s central and northern states where its occurrence is usually associated with single genetic lineages (Arroyo et al., 2011). Outbreaks of VS in the United States have historically occurred every 8–10 years with outbreaks often extending for 1–3 years after the first occurrence. Outbreak cycles usually initiate in the border states of Arizona, New Mexico, or Texas and spread as far north as Wyoming (Rainwater-Lovett et al., 2007; Perez et al., 2010). Phylogeographic studies indicate that epidemic VSNJV strains affecting the United States are monophyletic lineages emerging from enzootic progenitors circulating in southern Mexico (Rodriguez et al., 2000; Rainwater-Lovett et al., 2007; Velazquez-Salinas et al., 2014).

Recently, we provided a detailed description of the emergence and progressive northward migration of a particular VSNJV lineage, termed 1.1, which spread through central and northern Mexico between 2006 and 2009 and into the southern United States in 2012. Phylogenetic characterization based on the hypervariable region of the P gene strongly suggested that the latest common endemic ancestor of lineage 1.1 was lineage 1.2, a group of viruses confined in the endemic states of Veracruz and Tabasco (Velazquez-Salinas et al., 2014). However, the inherent molecular and biological differences between epidemic and endemic VSV strains remain poorly understood.

The aim of the current study was to examine the molecular and biological characteristics of an epidemic emerging strain of VSV (NJ0612NME6) compared to that of a closely (genetically) related endemic strain (NJ0806VCB). We conducted a comprehensive genomic analysis to determine relevant nucleotide and amino acid substitutions associated with the genetic divergence between the lineages. Furthermore, using a well-established model for pathogenesis studies in domestic pigs (a natural, vertebrate host of VSV) we sought to determine the biological differences between these two lineages. The findings of this study are discussed regarding mechanisms of viral pathogenesis associated with epidemic lineages of VSV, as well as their potential impact during epizootics of VSV.

## Materials and Methods

### Viral Strains

Two VSNJV strains, each representing one of the two genetic lineages, were used for this study. Viral strain NJ0806VCB, the lineage 1.2 representative virus, was isolated from a naturally infected bovine in Veracruz in 2006, where VSNJV occurs endemically (Arroyo et al., 2011). Viral strain NJ0612NME6, the lineage 1.1 representative virus, was isolated from a naturally infected equine in New Mexico during the 2012 VSNJV outbreak in the United States. Both viruses were obtained as first passage in Vero cells and were propagated once at an MOI of 0.01 TCID_50_ in the same cell line to produce high titer viral stocks that were stored at -70°C until usage. Viral strain VSIV-IFN-βb-NIS, which constitutively expresses human interferon beta (Naik and Russell, 2009) was used as a positive control for *in vitro* cytokine expression experiments and was kindly provided by Dr. Shruthi Naik.

### Cell Lines

The Vero (Vervet Monkey Kidney Epithelial cells) and BHK-21 (baby hamster kidney cells) cell lines were obtained from ATCC (ATCC catalog numbers CCL-81 and CCL-10, respectively). Primary fetal swine kidney cell cultures were obtained from the Foreign Animal Disease Diagnostic Laboratory (FADDL) at the Plum Island Animal Disease Center (PIADC), Greenport, NY, United States. Porcine peripheral blood was used to derive primary swine macrophage cell cultures as previously described (Zsak et al., 1996).

### Phylogenetic Analysis

Full-length genomic sequences of NJ0806VCB (accession #MG552608) and NJ0612NME6 (accession #MG552609) were obtained by the Sanger method as previously reported (Velazquez-Salinas et al., 2018). The evolutionary history of lineage 1.1 as well as its relationship with previously determined VSNJV strains affecting the United States and Mexico (Velazquez-Salinas et al., 2017a) was determined by phylogenetic analysis using relevant whole genome sequences available in GenBank. The genetic relationships were inferred using the MEGA 7 software package (Kumar et al., 2016) under the Maximum Likelihood optimality criterion with the General Time Reversible model and allowing some sites to be evolutionary invariable (Nei and Kumar, 2000). To evaluate the robustness of the tree, we applied a bootstrap analysis with 1,000 replicates. Additionally, synonymous (dS) and non-synonymous (dN) pairwise distance analyses were carried out using the program Sequence Distances in the SSE software version 1.2 (Simmonds, 2012).

### Amino Acid Substitution Analysis

The probability of a biologically meaningful amino-acid replacement occurring in the protein alignment was assessed using the Blocks Substitution Matrix (BLOSUM80). Positive scores imply a favored change; a zero score indicates a neutral change, and negative scores suggest a disfavored change (Henikoff and Henikoff, 1992; Betts and Russell, 2003).

### *In vitro* Growth Characterization

*In vitro* growth characteristics of NJ0612NME6 and NJ0806VCB were evaluated using multistep growth curves. Primary fetal swine kidney cell cultures and primary swine macrophage cultures were infected in triplicate at an MOI of 0.01 TCID_50_ per cell. Viruses were absorbed for 1 h (time zero) and samples were collected at 0, 4, 8, 24, 48 h post-infection (hpi). Titrations were conducted in BHK-21 cells as previously described (Martinez et al., 2003). Briefly samples were serially 10-fold diluted and added to the cells (suspension) in octuplicate wells and incubated at 37°C for 72 h. Titers, expressed as TCID_50_/ml, were calculated using the Reed and Muench method (Reed and Muench, 1938).

In addition, the ability of the two viral strains to grow at different temperatures was assessed by titrating high titter stocks of each virus at 32°C, 37°C, and 39°C using preformed monolayers of Vero cells. The ability of each virus to grow at different temperature conditions was quantified as the titer of the virus at either 32°C or 39°C divided by the titer of the virus at 37°C (Thermostability index). Values >1 reflect higher thermostability, values <1 reflect lower thermostability and values equal to 1 no changes in thermostability. Experiments were performed in triplicate.

### Transcriptional Regulation of the Immune Response

To evaluate the ability of each virus to regulate the antiviral response during *in vitro* infection, we used a previously described model based on the ability of VSV to disrupt the transcriptional wave of innate response genes in infected cells (Stojdl et al., 2003). Gene expression was determined in infected (MOI of 10 TCID_50_) primary fetal swine kidney cell cultures (FPKC) and primary swine macrophage cultures (SMC). At 5 hpi, total cellular RNA was extracted from mock-infected and infected cells. Steady state levels of mRNA accumulation were determined for 11 swine genes representative of the primary (IFN- β), secondary (IRF7, STAT 2, Mx1, OAS1, and PKR), and tertiary (IFN-α-1, IFN-α-7/11, IFN-α-9, IFN-α-10, and IFN-α-17) transcriptional waves of innate response genes. Analyses were done by quantitative reverse transcription real-time polymerase chain reaction (qRT-PCR) followed by a melting curve analysis as previously described (Borca et al., 2008). A change in the normalized mRNA expression level of a gene in VSV infected cells was deemed significant if it deviated from its respective expression level in mock-infected cells by at least threefold either up or down (Brukman and Enquist, 2006; Borca et al., 2008). As a control, we included a previously described recombinant VSIV engineered to express human IFN beta and the sodium iodide symporter (NIS) as this virus is unable to disrupt the innate immune response in normal cells (Naik and Russell, 2009).

### Animal Experiments

Pigs, a natural host of VSV, were used as a model of animal infection. The high susceptibility of this species to VSV has been well established providing an excellent model for pathogenesis studies (Clarke et al., 1996; Howerth et al., 1997; Stallknecht et al., 2001; Martinez et al., 2003).

Studies were conducted under an experimental protocol approved by the institutional animal care and use committee (IACUC protocol #245-05-14R) in a Biosafety Level 3-agricultural (BSL-3Ag) facility at the United States Department of Agriculture’s Plum Island Animal Disease Center (PIADC). Two replicate *in vivo* experiments were performed; one with each representative virus. In each of the experiments, eight Yorkshire pigs (8–10 weeks old and 25–30 kg in weight) were housed in the same isolation room. Within the room, pigs were divided into two groups (inoculated and contact) of four pigs each separated by double fencing to avoid direct contact. After a 1 week acclimation period, four pigs were intradermally inoculated in the snout with either NJ0612NME6 or NJ0806VCB as previously described (Martinez et al., 2003). Briefly, an intramuscular injection of a mixture of xylazine, ketamine, and telazol (4, 8, and 3 mg/kg, respectively) was used to sedate the animals. The epidermis of the snout was pricked 20 times using a dual-tip skin test applicator (Duotip-Test: Lincoln Diagnostics, Decatur, III) and 10^7^ TCID_50_ of virus in 50 μl of Dulbecco Modified Eagle Medium (DMEM) was placed on the scarified area. The area under the inoculum was re-sensitized by repeating the scarification procedure and the snout was held stationary in an upright, flat position for 3 min to assure sufficient contact time. The inoculated animals were kept separate from the contact group by double fencing through 24 h. The fencing separating the groups was subsequently removed and the contact pigs were allowed to co-mingle with the inoculated group for the duration of the experiment (21 days).

### Sampling and Clinical Evaluation

Throughout each of the experiments, sample collection and clinical evaluations were performed following a standardized protocol. Animals were sampled and clinically evaluated daily from days 0 through 10 and again at 14 and 21 days post-infection (dpi). Collected samples included whole blood, serum, oropharyngeal (OP) swabs and nasal swabs. Blood samples were collected from the jugular vein into EDTA-containing tubes (Vacutainer) or into tubes without anticoagulant to obtain serum. OP swabs were collected by directly targeting the tonsil of the soft palate using a large cotton swab while nasal swabs were collected by swiping small cotton swabs within the external nares. Directly after collection, all swabs were immersed in 2 ml minimal essential media containing 25 mM HEPES. The fluid absorbed by the large OP swab was extracted using an additional, brief centrifugation step. After initial processing, samples were immediately stored at -70°C until testing.

The severity and dissemination of the disease was evaluated using a clinical scoring system based on the location and distribution of the vesicular lesions. In brief, each of the 16 digits with a characteristic lesion contributed two points toward a cumulative score, with a single additional point added for vesicular lesions observed on the snout of the directly inoculated animals or two points for snout lesions on contact animals, two points added for lesions in the lower lip, oral cavity and on carpal/tarsal skin, thus allowing a maximum score of 45 and 46 for direct inoculated and contact pigs, respectively. Rectal temperatures were measured daily before sample collection.

### Post-mortem Sample Collection

Four pigs (two inoculated and two contact-exposed) representing the individuals with the highest accumulated clinical score from each of the two experiments, were euthanized for tissue harvest at 21 dpi by deep sedation (see above) followed by exsanguination. Tissues collected included: anterior tongue epithelium (ATONG), tonsil of the soft palate (PTON), nasopharyngeal tonsil (NTON), neck skin (NS), submandibular lymph node (SMLN), liver (LIV), spleen (SPL), snout skin (SNT), gastrohepatic lymph node (GHLN), coronary band-vesicle (CB-V), parotid lymph node (ParLN), and right popliteal lymph node (R-PopLN). Each tissue sample was divided into two 30 mg aliquots that were placed in individual tubes and frozen at -70°C until further processing.

### Viral RNA Detection

RNA extraction was carried out using Ambion’s MagMax-96 Viral RNA Isolation Kit (Ambion, Austin, TX, United States) on a King Fisher-96 Magnetic Particle Processor (Thermo Scientific Waltham, MA, United States) following a protocol previously described (Arzt et al., 2010). RNA (2.5 μl) was analyzed by real-time RT-PCR (rRT-PCR) targeting the VSNJV nucleocapsid gene (N), following a protocol previously described (Scherer et al., 2007). The only difference compared to the previously published protocol was a single nucleotide change introduced in the forward primer (5′-GCACTTCCTGATGGGAAATCA-3′) to match the sequence of the two viruses used in the current study. Reactions were performed with an ABI 7000 system (Applied Biosystems, Austin, TX, United States). **Supplementary Figure S1** shows the test sensitivity for the detection of these two strains. Cycle threshold values were converted into RNA genome copy numbers per 2.5 μl of RNA by use of standard curves based on analysis of 10-fold dilutions of *in vitro* synthesized VSNJV N RNA.

### Virus Isolation

Debris and potential bacterial contamination were cleared from aliquots of macerated tissue samples and fluid from nasal and OP swabs via centrifugation through 0.45 μm Spin-X filter columns (Costar cat. No 8163). The resulting fluids were diluted 1:5 in cell culture medium and 500 μl of each dilution applied to Vero cell monolayers in 24 well plates and observed for cytopathic effect (CPE) for 72 h. All CPE positive samples were confirmed as VSNJV utilizing rRT-PCR and titrated at 37°C in 96-well plates with preformed monolayers of BHK-21 cells. Each sample was tested in eight replicate wells. Titers were calculated as described above.

### Neutralizing Activity of Non-immune Porcine Serum

To test the non-specific inhibitory effect of pig serum on VSNJV, non-immune porcine serum (NPS) was collected from five healthy donor pigs, pooled and frozen at -70°C until used. A portion of the NPS was heat inactivated at 56°C for 30 min (NPSH). For neutralization assays, NJ0612NME6 was titrated in 10-fold dilutions in phosphate buffered saline solution (PBS) and 5 μl of each dilution incubated with 100 μl of either NPS or NSPH at 37°C for 1 h (Tesfay et al., 2014). As a control, viral dilutions were incubated with PBS only. After the incubation period, the reaction mixtures were used to infect monolayers of Vero cells and then overlaid with gum tragacanth and incubated at 37°C for 48 h. Finally, plaques were visualized by staining with crystal violet and viral titers were determined and expressed as plaque forming units (PFUs). All virus neutralization assays were conducted in triplicate.

### Complement Determination in Pig Serum

The number of complement units in pig serum was determined by a standard hemolytic assay following a previously described protocol (Kabat and Mayer, 1961). Pig sera were twofold diluted in gelatin veronal buffer (GVB) and 100 μl of each dilution was incubated with 200 μl of a sheep erythrocytes suspension (SES) (5% suspension with GVB previously sensitized with a rabbit antibody against sheep erythrocytes, kindly supplied by APHIS-FADDL) at 37°C for 1 h. Controls for the assay included: cell control (mixture of 100 μl of GVB and 200 μl of SES) and total lysis control (mixture of 100 μl of water and 200 μl SES). After incubation, mixtures were centrifuged at 931 × *g* for 10 min at 4°C and supernatants were read at A_541_ in a spectrophotometer. The percentage of hemolysis was calculated as follows: *y* = (A_541_ sample solution – A_541_ cell control)/A_541_ total lysis control. One hemolytic unit of complement (CH50 unit) was defined as the highest serum dilution that produced 50% of hemolysis.

### Antiviral Activity in Serum

Antiviral activity in serum was assessed using the Mx-CAT reporter assay (Fray et al., 2001), as previously described (Perez-Martin et al., 2012; Fernandez-Sainz et al., 2015). Briefly, MDBK-T cells were seeded in 24 well plates and after 24 h, 0.1 ml of either serum or specific amounts of recombinant human IFN-α 2A (1.95–1,000 U/ml) (standard control) were added to the respective wells. After 24 h of incubation at 37°C, cells were lysed and CAT expression was determined using a commercially available ELISA kit (Roche Applied Sciences, Indianapolis, IN, United States). Results were expressed as a unit of antiviral activity per ml. Previous studies have shown that this test measures type-I IFN activity (Fray et al., 2001; Francois et al., 2005).

### Detection of Cytokines in Serum of Infected Animals

Levels of total tumor necrosis factor (TNF), a previously characterized cytokine with antiviral activity against VSV (Mestan et al., 1988), and interleukin 6 (IL-6), a cytokine associated with immunosuppression activity by affecting the maturation of dendritic cells (Park et al., 2004), were determined by ELISA following the manufacturer’s protocol (R&D Systems, Minneapolis, MN, United States).

### Serum Neutralization Assay

Neutralizing antibodies against VSNJV were detected as previously described (Flanagan et al., 2001). Briefly, sera were collected and heat inactivated at 56°C for 30 min. Twofold serial dilutions of heat inactivated sera were incubated with 1000 TCID_50_ of VSNJV for 1 h at 37°C in 96-well plates, and after that Vero cells in a concentration of 1 × 10^6^ cells per plate were added in different plates. Plates were incubated at 37°C for 3 days. Serum neutralizing activity was reported as the reciprocal of the highest dilution giving 100% inhibition of CPE.

To estimate the levels of antibodies (IgM and IgG) during the acute phase of the disease, serum samples collected between 0 and 6 dpi were subjected to complement fixation assay (CFA). CFA was performed as previously described (Berninger et al., 2018). Briefly, control and test sera were diluted 1:5 and heat inactivated at 56°C for 30 min. Serial twofold dilutions of each sample were made in 96-well “U” bottom plates, followed by incubation at 37°C for 3 h in presence of complement and VSNJV antigen. A 1.4% solution of sheep red blood cells and hemolysin (rabbit anti-sheep red blood cells) was added and after a 30-min incubation at 37°C, the plates were read for the presence or absence of hemolysis. Endpoint titer was the last dilution showing hemolysis.

### Statistical Analysis

Statistical differences (*p* < 0.05) were calculated by the one unpaired *t*-test. This test compares the difference between means with the standard error of the difference, computed by combining the standard errors of the two groups. Each independent variable (inoculation days) was analyzed individually. Normal sample distribution was assessed by the Kolmogorov–Smirnov test. Calculations were performed using GraphPad Prism version 7.00 for Windows (GraphPad Software, La Jolla, CA, United States)^1^.

## Results

### Phylogenetic Analysis

**FIGURE 1:**
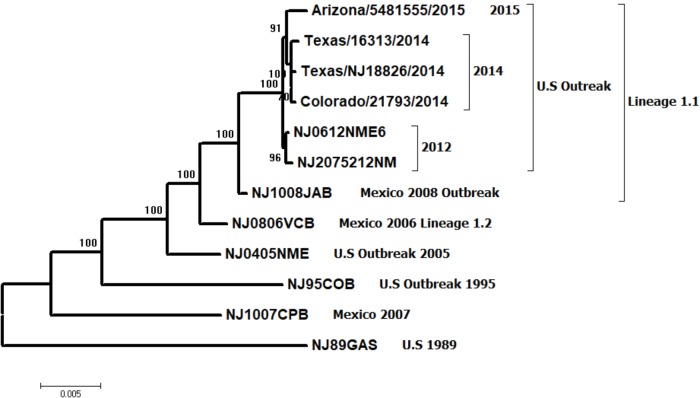
Maximum likelihood phylogenetic analysis of 13 VSNJV from Mexico and the United States, collected between 1989 and 2015 based on the P protein hypervariable region. Notice the relationship between the emergent lineage 1.1 and its closest endemic relative, lineage 1.2 (represented by viral strain NJ0806VCB). These viruses are distinct from previously characterized viral strains isolated during earlier outbreaks in the United States (1995 and 2005) and a viral strain collected in an endemic zone of Mexico during 2007. Numbers above the interior nodes represent bootstrap values obtained after 1,000 replicates.

To better understand the genetic relationship between viral strains NJ0612NME6 (epidemic lineage 1.1 representative virus from the United States) and NJ0806VCB (endemic lineage 1.2 representative virus from Mexico), a phylogenetic analysis was conducted using their full-length genomic sequences. Additional full-length genomic sequences of lineage 1.1 virus from Mexico (NJ1008JAB) and all available viruses from the United States outbreaks in 1995, 2005, 2012, 2014, and 2015 were included in the analysis. The NJ0612NME6 virus grouped with other lineage 1.1 viruses circulating in Mexico, as far back as 2006 and was ancestral to viruses detected in the United States in 2014 and 2015 (**Figure 1**). The basal branch position of viral strain NJ1008JAB suggests that the ancestral source of the viruses circulating in the United States were strains associated with lineage 1.1 previously circulating in central and northern Mexico between 2006 and 2009. After its emergence in the United States in 2012, lineage 1.1 differentiated into sublineages following geographic and temporal distribution but the closest relative to the ancestral NJ1008JAB was NJ0612NME6 (**Figure 1**). Our analysis confirmed that the closest endemic genetic relative to lineage 1.1 was lineage 1.2, which includes NJ0806VCB, and appeared in the phylogenetic tree as a basal branch to lineage 1.1. There was no direct phylogenetic relationship between lineage 1.1 and VSNJV lineages causing previous outbreaks in the United States, clearly indicating that the 2012 emergence of lineage 1.1 in the United States was the result of a new viral incursion from Mexico.

### Genomic Comparison

**FIGURE 2:**
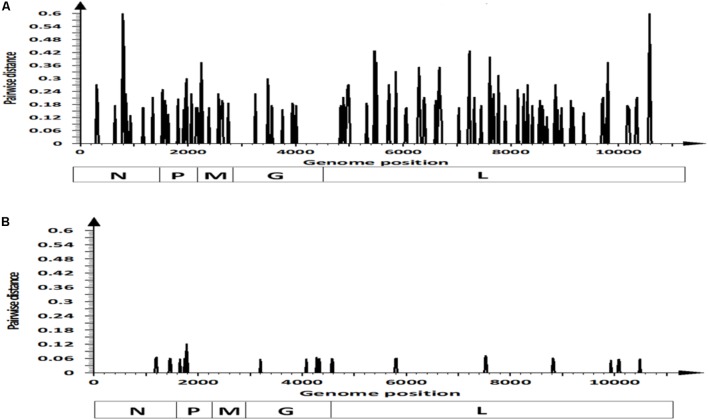
Pairwise distance analysis contrasting differences in: **(A)** synonymous and **(B)** non-synonymous sites within coding regions of viral strains NJ0612NME6 and NJ0806VCB. Bars in the graphics represent pairwise distance comparisons using 20 nt windows.

We analyzed nucleotide substitutions between viral strains NJ0612NME6 and NJ0806VCB to identify dS and dN substitutions. There were 111 nucleotide substitutions distributed across the coding regions of these two strains, with a majority of these substitutions (*n* = 88) being synonymous (**Figures 2A,B**). Overall, most of the 88 synonymous substitutions were located in the L gene (*n* = 56). Their distribution across previously described functional regions was as follows: N-terminal region (*n* = 3), conserved region (CR) I (*n* = 7), CR II (*n* = 2), CR IV (*n* = 7), CR V (*n* = 8), unstructured region (*n* = 9), CR VI (*n* = 6), and C-terminal region (*n* = 6), while eight dS substitutions were located in regions with non-specific functions. The remaining synonymous substitutions (*n* = 32) were distributed across the remaining genes as follows: M (*n* = 9), N (*n* = 8), P (*n* = 8), and G (*n* = 7). Only the substitutions in the P gene were located in known functional regions: domain I (*n* = 3), hypervariable region (*n* = 2), domain II (*n* = 1), and domain III (*n* = 1). Interestingly, of the 88 synonymous substitutions, 60.24% (*n* = 53) were consistently found in other viral strains associated within lineage 1.1 considered in this study, with 34.1% (*n* = 30) of them conserved among all viral strains isolated from the recent United States outbreak cycle (i.e., 2012–2015) (**Supplementary Figure S2**).

**FIGURE 3:**
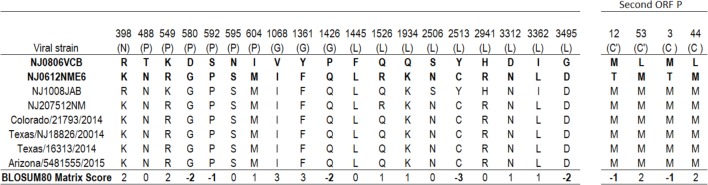
Amino acid differences between viral strain NJ0806VCB (1.2) and different viral strains from lineage 1.1. Numbers in the headings of each column represent the specific locations in the concatenated coding sequence of VSNJV, and letters in parenthesis denote the corresponding viral protein where the predicted substitution occurs. The BLOSUM80 matrix score at different positions reflects the biological meaningfulness of the amino acid substitution (see “Materials and Methods” section).

There were 23 predicted amino acid substitutions between NJ0612NME6 and NJ0806VCB, 19 of which were located in four of the structural proteins (N, P, G, and L) and the remaining four located in the C and C′ proteins encoded in the second reading frame of the phosphoprotein (**Figure 3**). The amino acid changes in the P protein were located in domain I (position 488) and the hypervariable region (positions 549, 580, 592, 595, and 604). Amino acid substitutions in the polymerase (L protein) were distributed across the N- terminal region (positions 1445 and 1526), CR II (position 1934), CR IV (position 2506), CR V (position 2513), unstructured region (position 2941), and C-terminal region (positions 3312 and 3495). The remaining four amino acid substitutions were located in regions of the N (*n* = 1) or G (*n* = 3) proteins with no described function.

Based on the Blosum80 matrix score, 47.9% (11/23) of the amino acid changes were classified as favorable, 21.7% (5/23) were classified as neutral and 30.4% (7/23) of the amino acid replacements (amino acid residues in the VSNJV genome at positions 580, 592, 1426, 2513, and 3495 and C′/C positions 12 and 3) resulted in predicted changes in size, charge, or hydrophobicity (**Figure 3** and **Supplementary Figure S3**).

### *In vitro* Growth Characterization

**FIGURE 4:**
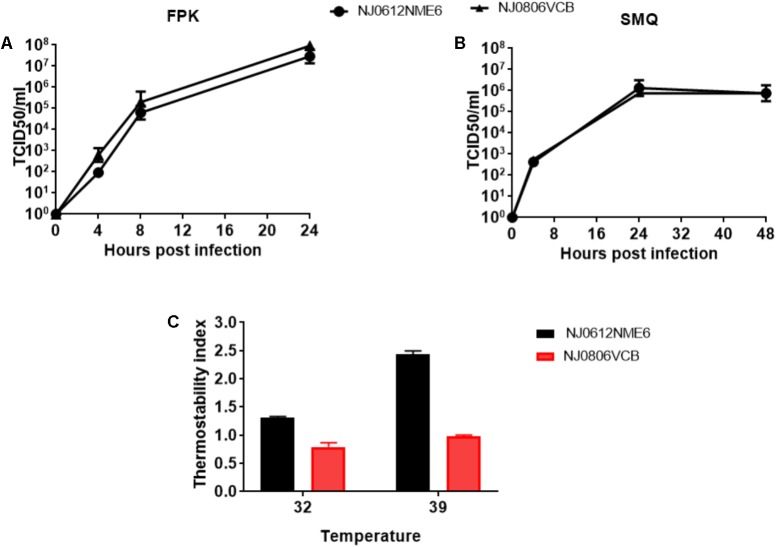
*In vitro* growth characteristics of NJ0612NME6 and NJ0806VCB. **(A)** Fetal porcine kidney cells and **(B)** porcine macrophage cells were infected at an MOI = 0.01 and virus yields obtained at the indicated times post-infection were titrated in Vero cells. Data represents the means and standard deviations from three independent experiments. **(C)** The ability of NJ0612NME6 and NJ0806VCB to grow at 32°C and 39°C. Thermostability index was determined by the viral titer of each strain at the specific temperature divided by the titer at 37°C. Values >1 reflect higher thermostability, <1 lower thermostability and =1 no changes in thermostability.

To explore possible biological differences between lineages 1.1 (NJ0612NME6) and 1.2 (NJ0806VCB) *in vitro* growth characteristics were evaluated in primary fetal swine cell cultures and primary swine macrophage cultures. Overall, both viruses displayed similar growth kinetics in both cell types (**Figures 4A,B**).

The ability of each strain to grow at two different temperatures (32°C and 39°C) was determined in Vero cells using a ratio of their titer at the given temperature divided by their titer at 37°C (Thermostability index). Overall, the results indicate that virus NJ0612NME6 was slightly more thermostable than virus NJ0806VCB (**Figure 4C**). The thermostability index of NJ0612NME6 (2.4 ± 0.08) was higher than that of NJ0806VCB (0.97 ± 0.031). The difference in thermostability indexes at 32°C was smaller, but NJ0612NME6 had a higher thermostability index (1.3 ± 0.02) than NJ0806VCB (0.79 ± 0.10) (**Figure 4C**).

### Transcriptional Regulation of the Immune Response

**FIGURE 5:**
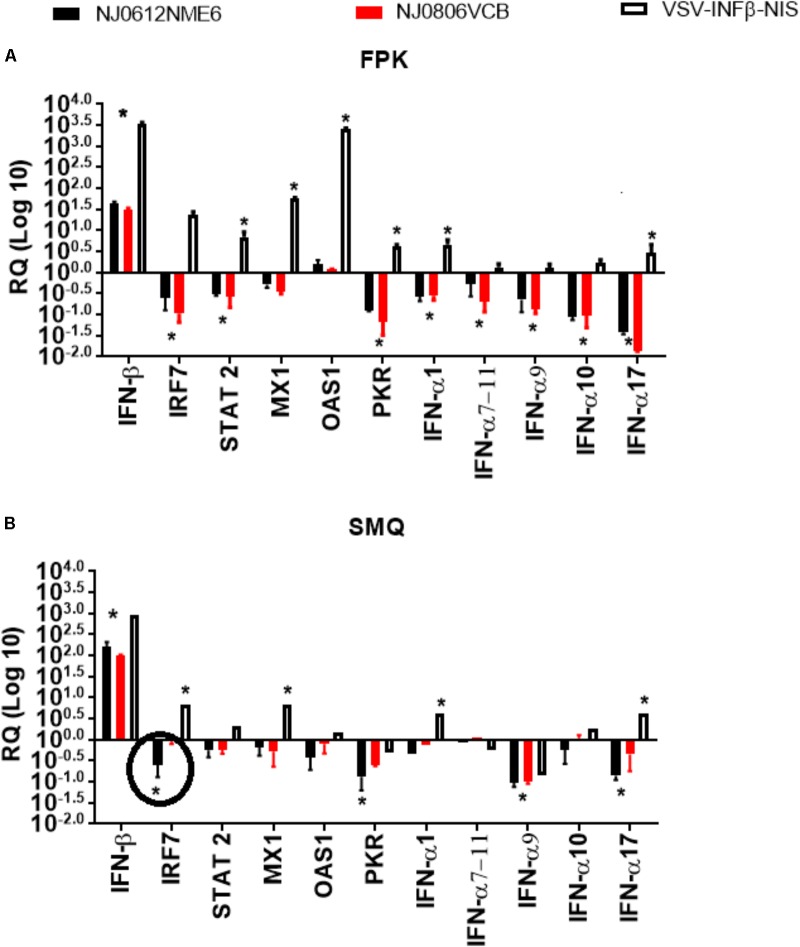
Transcriptional regulation of the immune response. Changes in gene expression measured by rRT-PCR were determined in **(A)** fetal porcine kidney cells (FPK) and **(B)** porcine macrophage cells (SMQ). VSV-INFβ-NIS was used as a positive control. Values are represented as relative quantities (RQs) of mRNA accumulation (estimated by 2^-ΔΔ*C*_T_^) with their corresponding SD. RQ values were considered significant when they departed from the corresponding level in uninfected cells by at least threefold in either direction. Asterisks represent significant values in one of the three viruses, and circles were used to represent significant differences between NJ0612NME6 and NJ0806VCB.

To evaluate the ability of each virus to regulate the antiviral immune response during *in vitro* infection, mRNA was extracted from infected cultures and the levels of 11 mRNAs were quantified by qRT-PCR to determine the ability of each strain to disrupt the transcriptional wave of innate immune gene responses during VSNJV infection (Stojdl et al., 2003). The change in normalized mRNA expression levels of a cellular gene in VSNJV infected cells was considered significant when it deviated at least threefold from its level in uninfected cells. A recombinant virus, VSIV-IFN-β-NIS, that constitutively expresses human interferon beta was used as a positive control for gene expression. In general, all three viruses stimulated the primary transcriptional wave of genes in both cell types as evidenced by the increased level of IFN-β mRNA upon infection (**Figures 5A,B**). As expected, VSIV-IFN-β-NIS induced a higher sustained level of IFN-β mRNA accumulation than the other viruses with no significant difference observed between NJ0612NME6 and NJ0806VCB. However, unlike VSIV-IFN-β-NIS, both NJ0612NME6 and NJ0806VCB disrupted the secondary and tertiary transcriptional waves of gene expression (i.e., responses independent of IFN-β protein production). This effect was more pronounced in primary fetal swine cell cultures where a large number of down-regulated cytokine genes were detected. Although both NJ0612NME6 and NJ0806VCB were able to disrupt the innate immune cytokine wave response, NJ0612NME6 was more efficient (3.17-fold) than NJ0806VCB in down regulating the transcription of IRF7 in primary swine macrophage cultures.

### Assessment of Lineage 1.1 and 1.2 Virulence in Swine

**FIGURE 6:**
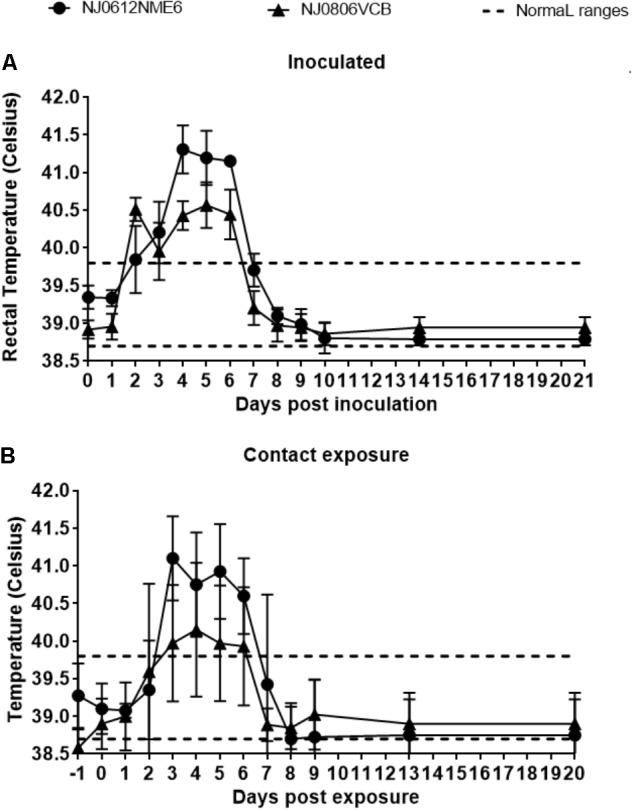
Fever response following NJ0612NME6 or NJ0806VCB inoculation. Two independent groups of four pigs were inoculated with 1 × 10^7^ TCID_50_ and then allowed to co-mingle with four contact pigs at 24 hpi. The rectal temperature of **(A)** inoculated and **(B)** contact exposure pigs is shown along with a dashed line which represents the standard rectal temperature of an uninfected pig. In all cases values represent means and standard deviations for each group of pigs.

**FIGURE 7:**
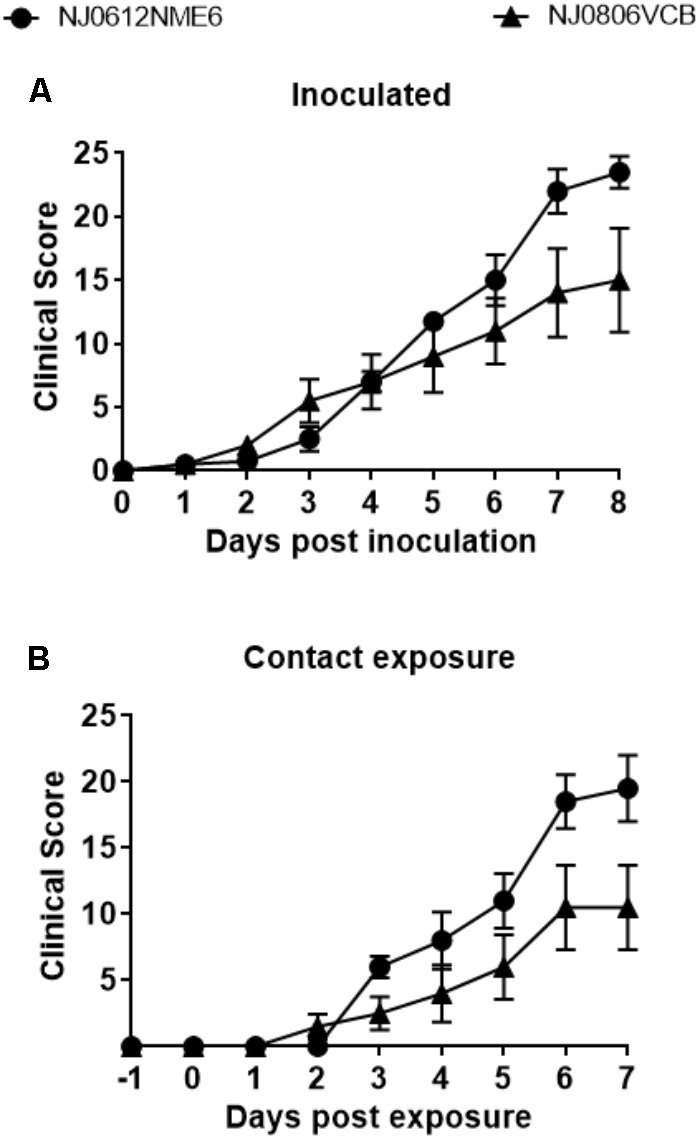
Clinical scores following NJ0612NME6 or NJ0806VCB inoculation. Two independent groups of four pigs were inoculated with 1 × 10^7^ TCID_50_ and then allowed to co-mingle with four contact pigs at 24 hpi. Pigs were examined daily for the presence of lesions in the snout, mouth, lips, feet, and the cumulative clinical scores are given for the **(A)** inoculated and **(B)** contact exposure pigs. In all cases values represent means and standard deviations for each group of pigs.

Following the experimental design described above, we utilized a previously established swine model (Martinez et al., 2003) to contrast the virulence of lineage 1.1 and 1.2 viruses. Increased body temperature (>39.8°C) was observed beginning at 2 days post infection (dpi) in directly inoculated or 3 days post exposure (dpe) in contact exposed animals and lasted until 6 dpi and 6 dpe for directly inoculated and contact exposed animals, respectively. Higher overall temperatures were observed in animals infected with NJ0612NME6 (lineage 1.1) than in those infected with NJ0806VCB (lineage 1.2) regardless of the route of infection (**Figures 6A,B**). Vesicles developed within the area of scarification in all directly inoculated pigs starting at 2 dpi for both viruses, subsequently increased in size and ruptured by 3–5 dpi. Clinical scores, based on the number of vesicular lesions in specified areas (digits, oral mucosa, lips, snout and the skin covering the carpal or tarsal joints) increased steadily from 3 to 4 dpi and peaked at 8 dpi for both viruses. Overall, average clinical scores were higher for directly inoculated (23.5 ± 2.5) and contact-exposed pigs (19.5 ± 2.5) infected with NJ0612NME6 than those observed for directly inoculated (15 ± 4.08) and contact-exposed pigs (10.5 ± 3.2) infected with NJ0806VCB (**Figures 7A,B**). Additionally, between 4 and 5 dpi an inflammatory response was observed in carpal and tarsal joints of all pigs infected with NJ0806VCB.

### Viral Shedding

**FIGURE 8:**
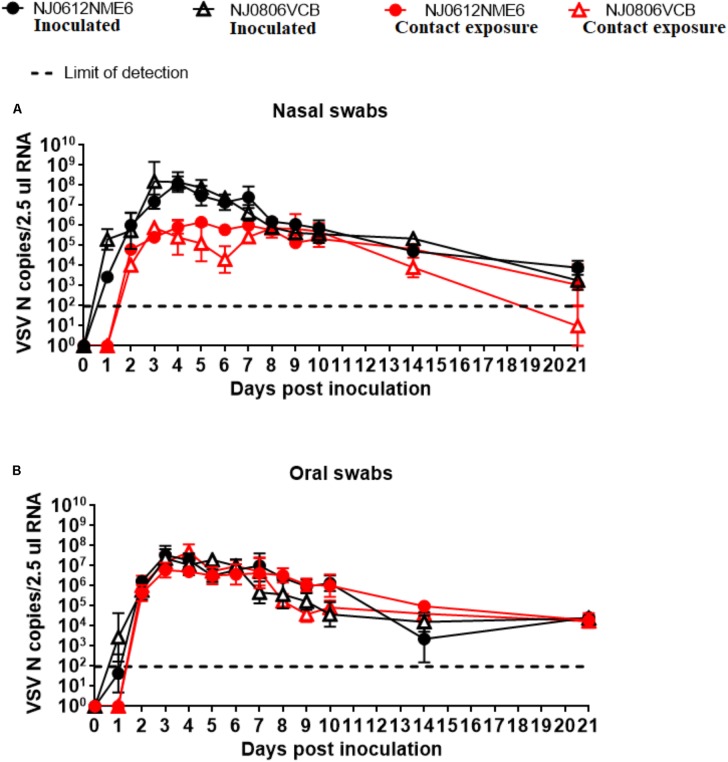
Detection of viral RNA in clinical samples. Biological samples were collected at indicated time points after inoculation with NJ0612NME6 or NJ0806VCB and RNA was analyzed by rRT-PCR to detect VSV nucleocapsid (N) RNA in **(A)** nasal swabs or **(B)** oral swabs of both directly inoculated and contact exposure pigs.

Viral dissemination and shedding was evaluated by rRT-PCR and virus isolation from nasal and OP swabs. No significant differences in genome copy number were found between groups of pigs infected with either virus. VSNJV RNA was detected in nasal and OP swabs as early as 1 dpi, peaked between 3 and 4 dpi, and remained at detectable levels until the end of the experiment in all pigs regardless of the virus or route of infection (**Figures 8A,B**). However, an average higher RNA copy numbers (∼100-fold) were observed in nasal swabs collected between 3 and 6 dpi from directly inoculated animals versus those infected by contact exposure, regardless of the virus used (**Figure 8A**).

**FIGURE 9:**
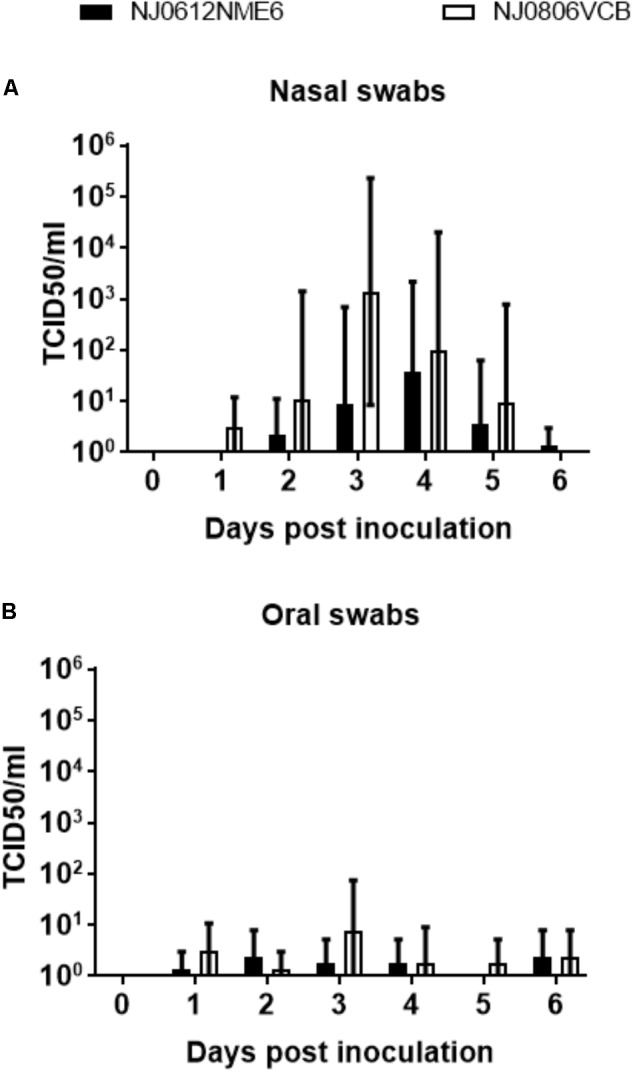
Detection of infectious virus in clinical samples. Infectious virus was isolated in Vero cells from **(A)** nasal swabs or **(B)** oral swabs after infection with NJ0612NME6 or NJ0806VCB at expressed days post-infection. Positive isolations were confirmed by rRT-PCR and viral titrations were conducted in Vero cells. Viral titers are expressed in TCID_50_/ml.

Infectious virus was intermittently recovered from nasal and OP swabs of pigs infected with both viruses between 1 and 6 dpi. The detection of infectious virus from nasal and OP swabs was earlier and slightly higher in pigs infected by NJ0806VCB than in the ones infected by NJ0612NME6. Overall, higher titers of infectious virus were recovered from nasal swabs than from OP swabs in both groups regardless of the virus used for the infection (**Figures 9A,B**). For nasal swabs, the peak viral titers occurred between 3 and 4 dpi and reached maximum viral titers of approximately 5.5 TCID_50_/ml. This peak was consistent with the time when vesicles on the snout ruptured in directly inoculated animals, and might explain why the majority of positive viral isolations (92%), were from pigs infected by direct inoculation regardless of the virus (**Figure 9A**). Conversely, recovery of infectious virus from OP swabs was similar in all groups regardless of the route of exposure or the virus (**Figure 9B**).

### RNAemia

**FIGURE 10:**
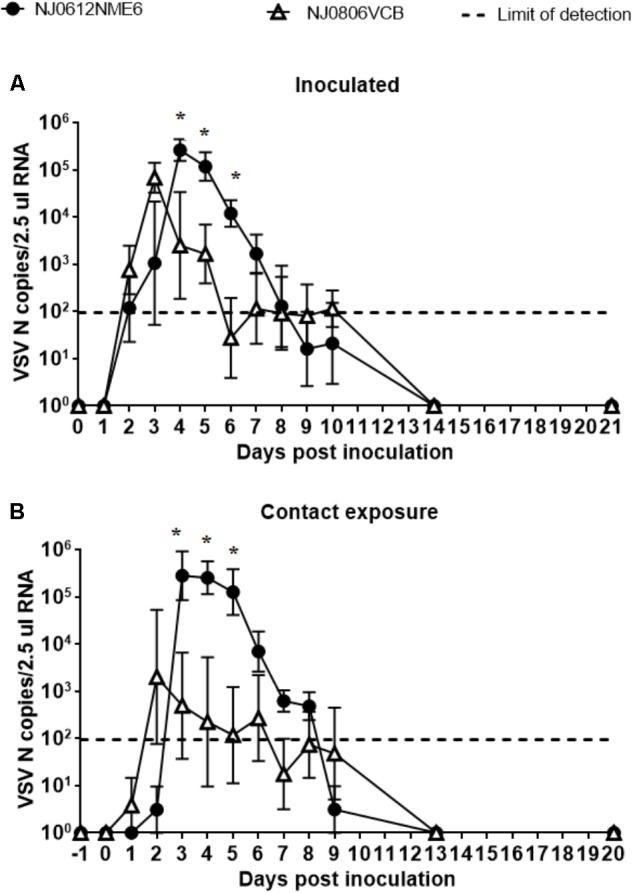
RNAemia. VSNJV RNA in blood was detected in **(A)** inoculated and **(B)** contact exposure pigs. Dashed lines represent the limit of detection of rRT-PCR (1.98 VSV N copies/2.5 μl RNA). Values represent means and standard deviations for each group, and significantly different values (*p* < 0.05) between groups are indicated with asterisks (^∗^).

To assess the presence of VSV in the bloodstream, blood samples were collected at multiple times post infection. Total RNA was isolated and evaluated by rRT-PCR to determine the number of viral genome copies. Viral RNA in blood (RNAemia) was first detectable at 2 dpi and peaked at 3 or 4 dpi depending on the viral strain (**Figures 9A,B**). In pigs infected with NJ0806VCB, RNAemia peaked earlier than in pigs infected with NJ0612NME6, regardless of exposure route. However, between 4 and 6 dpi, pigs infected with NJ0612NME6 (regardless of the route) had a significantly higher level of RNAemia (*p* < 0.05) relative to pigs infected with NJ0806VCB. Levels of RNAemia dropped gradually and fell below the limit of detection by 9 dpi (**Figures 10A,B**). Similar results were obtained using serum samples (not shown).

Despite the high levels of RNAemia in the blood and serum of infected pigs, we were unable to recover infectious virus. Multiple attempts of overlaying blood and serum samples on susceptible BHK-21 or Vero cells with varying dilutions of the blood or serum, in order to dilute out potential inhibiting factors, failed to yield infectious virus.

### Neutralization of VSV by Non-immune Pig Serum

**FIGURE 11:**
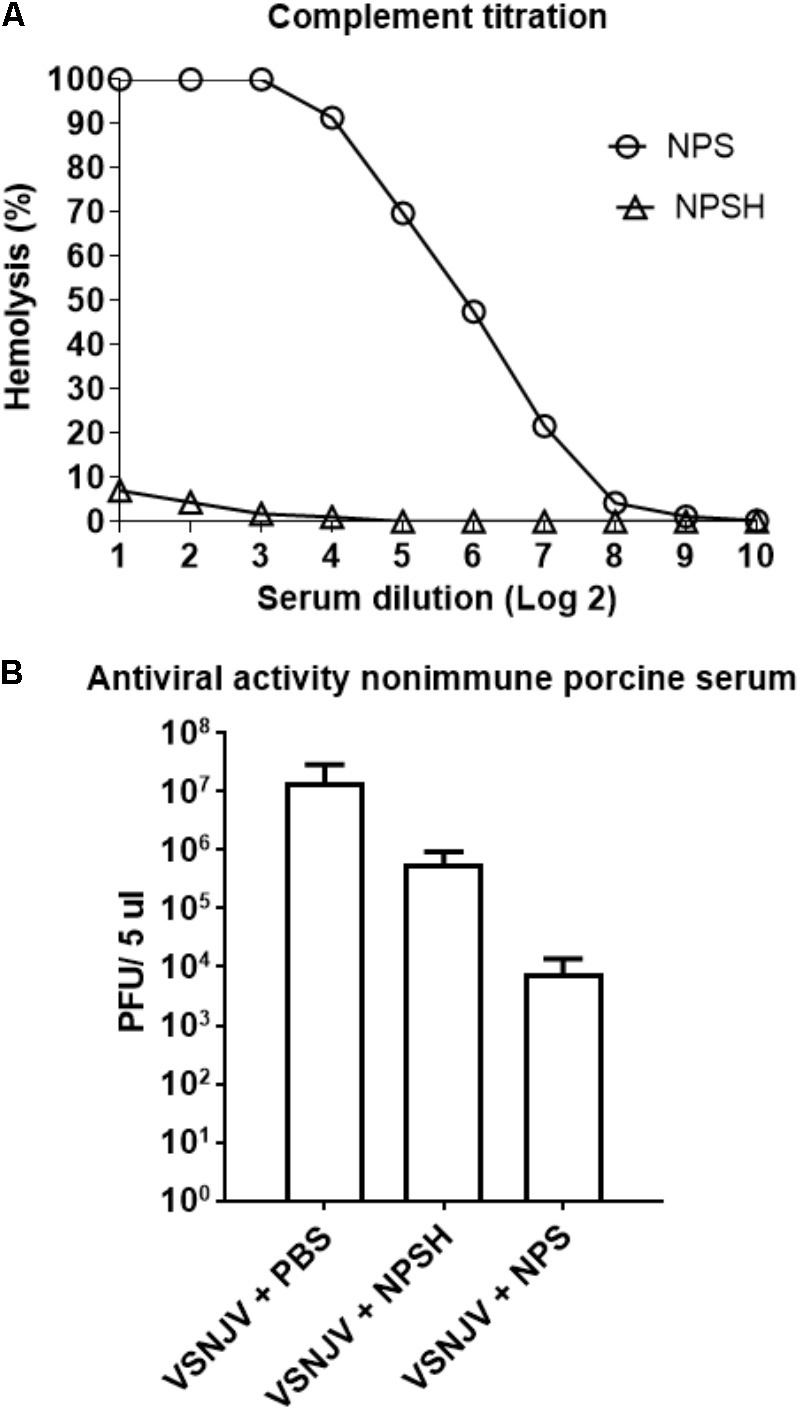
Non-immune porcine serum (NPS) neutralizing activity. **(A)** The number of complement units in NPS and heat-inactivated NPS (NPSH) were determined by standard hemolysis technique. **(B)** The ability of NPS or NPSH to neutralize VSNJV infection was determined by plaque assay. As a negative control de virus was incubated with PBS.

Previous reports (Beebe and Cooper, 1981; Tesfay et al., 2013, 2014) suggest that non-immune serum from humans, mice and dogs neutralize VSV by the concerted actions of IgM and complement. To explore if this was also the case for pigs, we evaluated the neutralizing activity of non-immune pig serum (NPS) against VSNJV. First, the amount of complement present in NPS was determined using a standard hemolytic assay. For this determination, the NPS was used either fresh or heat inactivated (NPSH), a condition known to inactivate complement. We found 64 complement hemolytic (CH50) units/100 μl in NPS, while minimal complement activity (0.28 CH50 units/100 μl) was detectable in heat-inactivated NPS (NPSH) (**Figure 11A**). Once we determined the presence of complement in NPS, we tested the ability of both NPS and NPSH to neutralize NJ0612NME6. Results showed that the titer of NJ0612NME6 was reduced from 7.16 ± 0.1 to 3.89 ± 0.1 log_10_ PFU (1000-fold) after 1 h of incubation with NPS compared with the untreated control (VSNJV + PBS) (**Figure 11B**). Interestingly, despite the heat inactivation, NPSH was still able to reduce the infectious titer by 10-fold (from 7.16 ± 0.1 to 5.76 log_10_ ± 0.13 PFU) (**Figure 11B**). Therefore, the non-specific viral neutralizing activity mediated by the presence of a mix of thermostable and thermolabile factors (such as complement proteins) in pig blood explains in part the failure to isolate infectious VSNJV from infected pig blood and serum.

### Innate Immune Response

**FIGURE 12:**
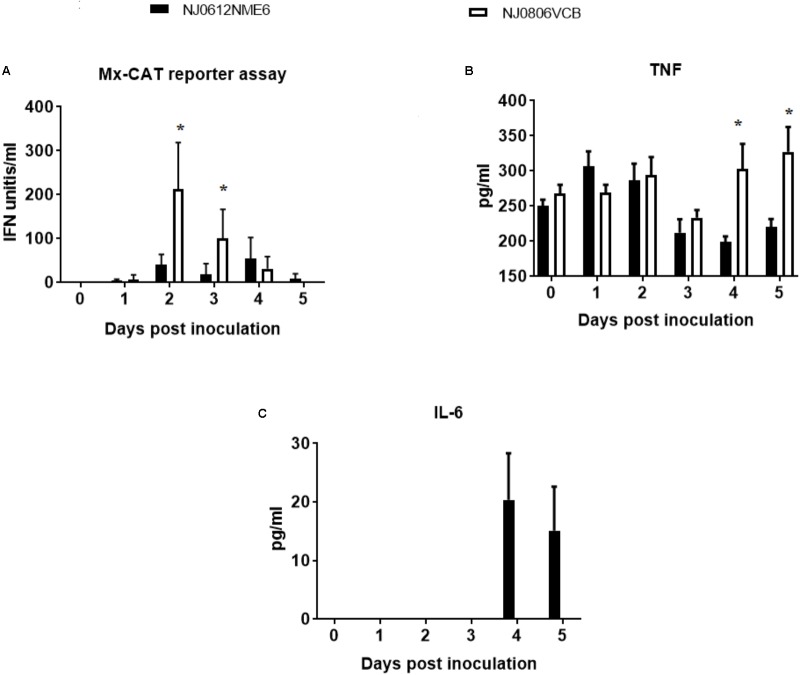
Innate immune response following infection in pigs with NJ0612NME6 or NJ0806VCB. Serum samples collected from inoculated during the acute phase of the infection was used to evaluate: **(A)** Type I IFN antiviral activity by MX-CAT reporter assay, **(B)** tumor necrosis factor (TNF), and **(C)** interleukin 6 (IL-6) by ELISA. Significantly different values (*p* < 0.05) between groups are indicated with asterisks (^∗^).

To assess the differences in the ability of NJ0612NME6 and NJ0806VCB to modulate the innate immune response *in vivo*, the antiviral activity and total levels of TNF and IL-6 in serum were analyzed in samples collected between 0 and 5 dpi. Serum type I interferon levels determined by Mx-CAT reporter assay showed significant (*p* < 0.05) higher levels of antiviral activity (∼82.03 and 171.81 IFN units/ml) in pigs infected with endemic virus NJ0806VCB between 2 and 3 dpi relative to pigs infected with epidemic virus NJ0612NME6 (**Figure 12A**). On the other hand, significant (*p* < 0.05) higher levels of total TNF (105.19 ± 45.40 pg/ml) were detected in pigs infected with NJ0806VCB between 4 and 5 dpi relative to pigs infected with NJ0612NME6 (**Figure 12B**). Conversely, IL-6 detectable levels (27.5 ± 8.81 pg/ml) in serum were only found in pigs infected with NJ0612NME6 between 4 and 5 dpi (**Figure 12C**) Overall, these results suggest that NJ0612NME6 has increased ability than NJ0806VCB to down regulate the innate immune response.

### Adaptive Immune Response

**FIGURE 13:**
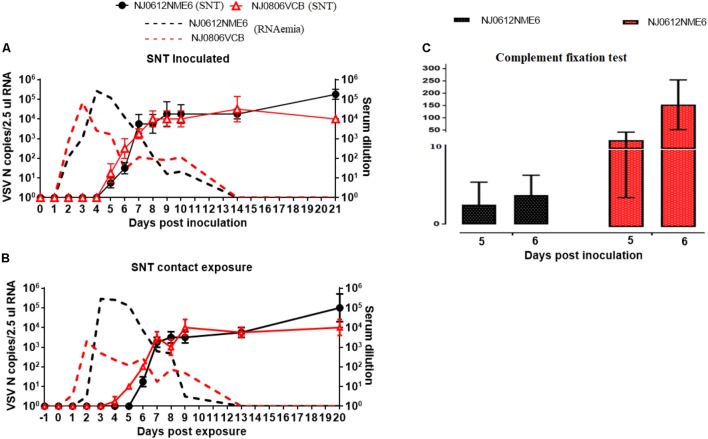
Adaptive immune response. Serum neutralization antibody titers following infection with NJ0612NME6 or NJ0806VCB. Adaptive immune response was monitored by measuring anti-VSV antibodies in serum collected at indicated the time points in **(A)** inoculated or **(B)** contact pigs. The broken lines in black and red represent levels of viral RNA in the blood of pigs infected with NJ0612NME6 and NJ0806VCB, respectively, indicating a negative correlation between antibody immune response and RNAemia phase. **(C)** The early antibody response in infected pigs with different viruses was evaluated by complement fixation test.

Neutralizing antibodies against VSNJV were first detected at 5 dpi in directly inoculated and 6 dpe in contact exposed animals. Antibody titers were similar in both groups at 14 dpi. However, at 21 dpi, neutralizing antibody levels were 10-fold higher in pigs infected with NJ0612NME6. Interestingly, while the decrease of RNAemia in pigs infected with NJ0806VCB occurred before the increase in the antibody titers, the decrease of RNAemia in pigs infected with NJO612NME6 was clearly associated with the development of the antibody response (**Figures 13A,B**). In addition, we assessed the early antibody response (IgM/IgG) in sera from animals inoculated with either virus using complement fixation test (**Figure 13C**). Interestingly, negative results were obtained in both groups of animals between 0 and 4 dpi. However, between 5 and 6 dpi a 10- and 100-fold higher titers of complement fixation antibodies was observed in pigs infected with NJ0806VCB than in those infected with NJ0612NME6.

### Post-mortem Examination

**FIGURE 14:**
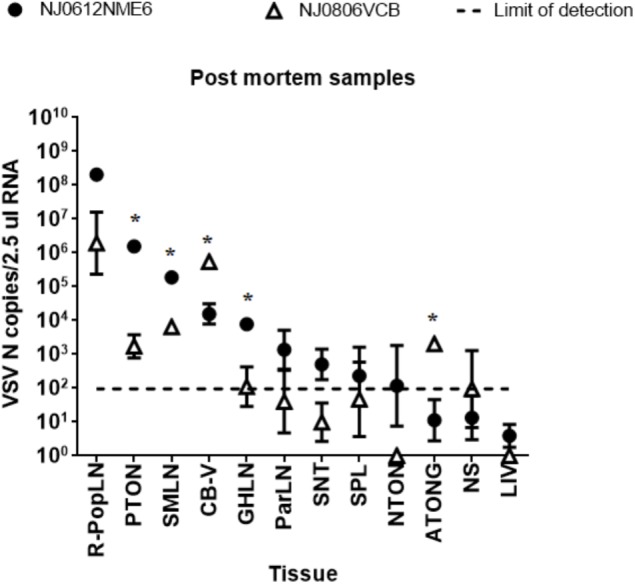
Detection of viral RNA in postmortem samples. RNA from tissues collected at 21 dpi was analyzed by rRT-PCR to detect VSV nucleocapsid RNA. The dashed line represents the limit of detection of rRT-PCR (1.98 VSV n copies/2.5 μl RNA). Values represent means and standard deviations for four pigs (two inoculated and two contact) from each group. Significant differences (*p* < 0.05) between groups are indicated by asterisks (^∗^).

To determine virus distribution in tissues from pigs infected with NJ0612NME6 or NJ0806VCB, 12 selected tissues were harvested from two directly inoculated and two contact-exposed pigs from each virus group at 21 dpi and 20 dpe, respectively. Viral RNA was detected in lymphoid and epithelial tissues from lesion sites for all pigs. The popliteal lymph node (Pop-LN) was the tissue with the highest levels of viral RNA in both groups (**Figure 14**). Overall, significant (*p* < 0.05) mean detection levels of viral RNA in lymphoid tissues (PTON, SMLN, and GHLN) was greater in pigs infected with NJ0612NME6 than in those infected with NJ0806VCB. Conversely, significantly (*p* < 0.05) higher levels of viral RNA in epithelial samples (CB-V and ATONG) were detected in pigs infected with NJ0806VCB. Interestingly, even with the high amount of VSNJV RNA found in most of these tissues, infectious virus was not recovered from any of the tissue samples collected post-mortem.

## Discussion

In this study, we determined distinctive molecular and biological characteristics associated with the increased virulence in pigs of an epidemic, emergent VSV viral strain from lineage 1.1 and contrasted these results to those obtained with a closely related endemic viral strain from lineage 1.2 (Velazquez-Salinas et al., 2014). These strains were compared using a combination of bioinformatics tools, various *in vitro* assays as well as *in vivo* pathogenesis experiments in pigs, a natural vertebrate host of this virus. Results indicated that these strains differ in their ability to cause disease in pigs, and that these differences might be related to the increased capability of epidemic strains to modulate the host innate immune response.

Our comparative analysis identified multiple genomic differences that might be related to the increased virulence of epidemic strain NJ0612NME6. A previous report showed that multiple substitutions in the Venezuelan equine encephalitis virus genome were responsible for the increased virulence associated with the emergence of epizootic strains (Greene et al., 2005).

The high number of dS substitutions observed within lineage 1.1 suggests that during the evolution of this epidemic strain, dS sites in the genome are evolving under positive selection. This pattern of evolution is consistent with populations of VSV adapting to specific conditions (Novella et al., 2004). Similar to our analysis, a previous study of natural populations of arboviruses supported the relevance of dS substitutions related to the specific codon usage in their respective hosts (Velazquez-Salinas et al., 2016). Furthermore, a previous study showed that the experimental introduction of dS substitutions in the L gene of VSV decreased virulence in mice (Wang et al., 2015). Comparing the differences in virulence between the two viral lineages tested in our study, a possible role of dS substitutions on viral virulence should be considered.

Very little is known about the role of specific amino acid replacements in VSV virulence in natural hosts. One of the few proteins known to influence VSV virulence is the M protein, which has been recognized as a major immune regulatory protein for its ability to suppress IFN-bβ gene expression (Ferran and Lucas-Lenard, 1997; Ahmed et al., 2003). However, our results showed identical deduced amino acid sequences for the M proteins between the lineages 1.1 and 1.2 representative viruses, indicating that other genomic determinants might be mediating virulence.

Of the 19 predicted amino acid substitutions located in the viral structural proteins of the epidemic strain, 18 were located in the proteins P, G, and L. Interestingly, despite the P protein constituting only ∼7.7% of the total coding sequence, almost half of the total predicted amino acid substitutions between the two viruses were located within this protein. Very little is known about the role of the P protein in VSV virulence. A recent study conducted with spring viremia of carp virus (another vesiculovirus) showed the ability of the P protein to regulate the immune cellular response by decreasing IFN production (Li et al., 2016). This is consistent with the evolutionary role of this protein to antagonize IFN activities in other Rhabdoviruses such as rabies virus (Rieder and Conzelmann, 2011; Okada et al., 2016) and makes the P protein an interesting target for future studies of VSV virulence.

The VSV G protein has been described as a determinant of virulence in swine (Martinez et al., 2003). Previous studies have shown the ability of the G protein to trigger type I IFN secretion *in vitro* (Georgel et al., 2007; Janelle et al., 2011). Interestingly, amino acid substitutions at residues 1068 (V→I) and 1361 (Y→F) reported in our study are in a region of G that influences IFN secretion (Georgel et al., 2007; Janelle et al., 2011). This might explain the decreased serum levels of systemic antiviral activity and the increased virulence in pigs infected with the epidemic strain.

Here, we report the ability of lineage 1.1 (NJ0612NME6) to produce a higher number of vesicular lesions in pigs relative to NJ0806VCB, its closest endemic relative. Our results support previous hypotheses that differences in severity of clinical signs may have a direct impact on viral spread during epidemics by increasing animal-to-animal transmission as well as virus availability for insect-vectored transmission (Smith et al., 2012). This is also consistent with previous studies highlighting the importance of vesicular lesions as a primary source of infectious virus during transmission (Stallknecht et al., 2001). Another differential clinical finding was the ability of NJ0612NME6 to induce a higher febrile process relative to NJ0806VCB. Interestingly, *in vitro* replication of VSV at temperatures above 40°C represents a selective factor favoring the emergence of variants with an increased capacity to evade the antibody neutralization activity by polyclonal sera of VSV infected animals, suggesting the ability of VSV to avoid the immune repose during febrile periods (Presloid et al., 2016). This is an interesting concept considering our *in vitro* results that suggested that lineage 1.1 has a slightly increased ability to grow at higher temperatures than lineage 1.2. This ability could be linked to amino acid differences between both strains in the G protein. Moreover, it might help to explain the increased clinical scores observed in pigs infected with virus NJ0612NME6 despite their higher febrile response compared to pigs infected with the endemic virus. This observation might encourage further studies aimed at understanding the role of specific residues in the G protein relative to virus thermostability and how increased thermostability might impact viral spread during epidemics.

Importantly, this is the first report demonstrating the presence of viral RNA in serum and blood samples of pigs experimentally infected with VSV. This finding is consistent with previous reports describing virus RNA in cattle and horses naturally infected with VSV (Tolardo et al., 2016), and horses experimentally infected with VSIV (Howerth et al., 2006). In fact, VSV RNA was regularly detected in serum samples from cattle naturally infected in Mexico with a viral strain associated with lineage 1.1 (Velazquez-Salinas, unpublished data).

Although previous reports indicate the absence of viremia during experimental VSV infection of pigs (Comer et al., 1995a,b; Stallknecht et al., 1999), our results suggest the existence of a viremic phase during the acute infection of pigs that might be masked by the inhibitory effect of heat stable and thermolabile serum proteins. Despite our inability to isolate infectious virus from blood and serum, our results indicated that the higher levels of viral RNA found in the blood of pigs infected with NJ0612NME6 correlated well with higher fever and clinical scores relative to pigs infected with NJ0806VCB, and suggest that differences in blood RNA levels during the acute phase of the infection might be used as a virulence marker in future pathogenesis studies in pigs.

We showed that infection of pigs with an epidemic or endemic VSV induced disparate serum levels of IgM/IgG antibodies, systemic antiviral activity, TNF and IL-6 during the acute phase, as well as marked differences in virulence. The earlier development of antibody response (determined by CFA) in pigs infected with virus NJ0806VCB compared to those infected with virus NJ0612NME6 constitutes an important finding in our study and might help to understand differences in virulence. The role of IgG/IgM antibodies in serum of different species have been shown to play an important role in neutralizing VSV by activating the classical complement pathway (Beebe and Cooper, 1981; Tesfay et al., 2013, 2014). One possible explanation for the faster IgM/IgG response seen in pigs infected with NJ0806VCB might be associated with the higher type I IFN response observed in these pigs, since type I IFN has been previously associated with the increase of primary antibody responses during VSV infections in a mice model (Fink et al., 2006; Fuertes et al., 2013).

The decreased levels of antiviral activity, attributed to type I IFN, in pigs infected with the epidemic strain provide a plausible explanation for the increased virulence observed in this strain, as type I IFN has been shown to have a key role in clearing VSV infection in mice (Muller et al., 1994). A recombinant VSV expressing human interferon beta was shown to be fully attenuated in experimental infection of healthy pigs, promoting the expression of multiple interferon stimulated genes (Velazquez-Salinas et al., 2017b). Our *in vitro* pathogenesis results showed that epidemic VSV was able to down-regulate the transcription of IRF-7, considered a master up-regulator of the interferon response (Ning et al., 2011). This provides a possible mechanism for the decreased antiviral activity detected during *in vivo* studies. Unfortunately under the conditions of our experiments and based on the intrinsic ability of VSV to interrupt the translation in the cell by blocking export of mRNA to the cytoplasm, we couldn’t appreciate the biological effects produced by the down regulation of IRF-7 observed in our study.

On the other hand, the decreased levels of TNF in pigs infected with the epidemic VSV may also contribute to the increased virulence. This cytokine can induce the expression of interferon stimulated genes independently of the known mechanism described by the IFN JAK-STAT cascade (Wang et al., 2016). A previous study showed that systemic circulation of this cytokine in infected mice with VSV results in activation of the innate and adaptive immune responses, and this plays a critical role for survival (Shinde et al., 2018). Furthermore, cell cultures previously treated with TNF were able to induce antiviral activity against VSV in a dose dependent manner (Mestan et al., 1988).

Finally, the increased levels of IL-6 found in the serum of pigs infected with the epidemic strain might explain the higher body temperatures observed in these pigs. This cytokine is a potent endogenous pyrogen, and its systemic circulation in patients infected with other arboviruses like Chikungunya virus and Crimean-Congo hemorrhagic fever virus, has been associated with the infection of highly virulent strains. In fact, IL-6 is considered a biomarker of virulence during the infection with these viruses (Ergonul et al., 2006; Ng et al., 2009). A previous study in mice showed that IL-6 plays a key role regulating the differentiation of dendritic cells *in vivo*, inhibiting the T-cell immune response (Park et al., 2004). The ability of IL-6 to negatively affect the T-cell response might be related to the increased levels of virulence observed in pigs infected with the epidemic strain. Therefore, it is possible that differential levels of IFN, TNF and IL-6 among pigs infected with the epidemic and endemic strains may explain the increased virulence observed with the epidemic strain.

## Conclusion

Our results showed important molecular and biological differences between endemic and epidemic strains of VSV. These differences include the epidemic strain’s increased ability to modulate innate immune responses during infection and its increased virulence compared to the endemic strain. The increased virulence in the vertebrate host could increase virus availability for animal-to-animal and vector transmission and might help explain the successful spread of this strain into non-endemic regions.

## Author Contributions

LV-S, SP, CS, JP, MB, AV-R, JA, and LR conceived and designed the experiments. LV-S, SP, and EO performed the experiments. LV-S, SP, and LR analyzed the data. LR, MB, and JA contributed the reagents, materials, and analysis tools. LV-S, SP, CS, MB, AV-R, JA, and LR wrote the manuscript.

## Conflict of Interest Statement

The authors declare that the research was conducted in the absence of any commercial or financial relationships that could be construed as a potential conflict of interest.

## Footnotes

1 www.graphpad.com

## Supplementary Material

FIGURE S1 Equal sensitivity of rRT-PCR to detect VSV viruses 1.1 and 1.2.

FIGURE S2 Synonymous substitution pattern on different genes associated with viral lineage 1.1.

FIGURE S3 Amino acid chemical properties.
