# Supplementary figures and images for: Increased Virulence of an Epidemic Strain of Vesicular Stomatitis Virus Is Associated With Interference of the Innate Response in Pigs
Source: Front Microbiol. 2018 Aug 15;9:1891. doi: 10.3389/fmicb.2018.01891 (PMC6104175; doi:10.3389/fmicb.2018.01891)

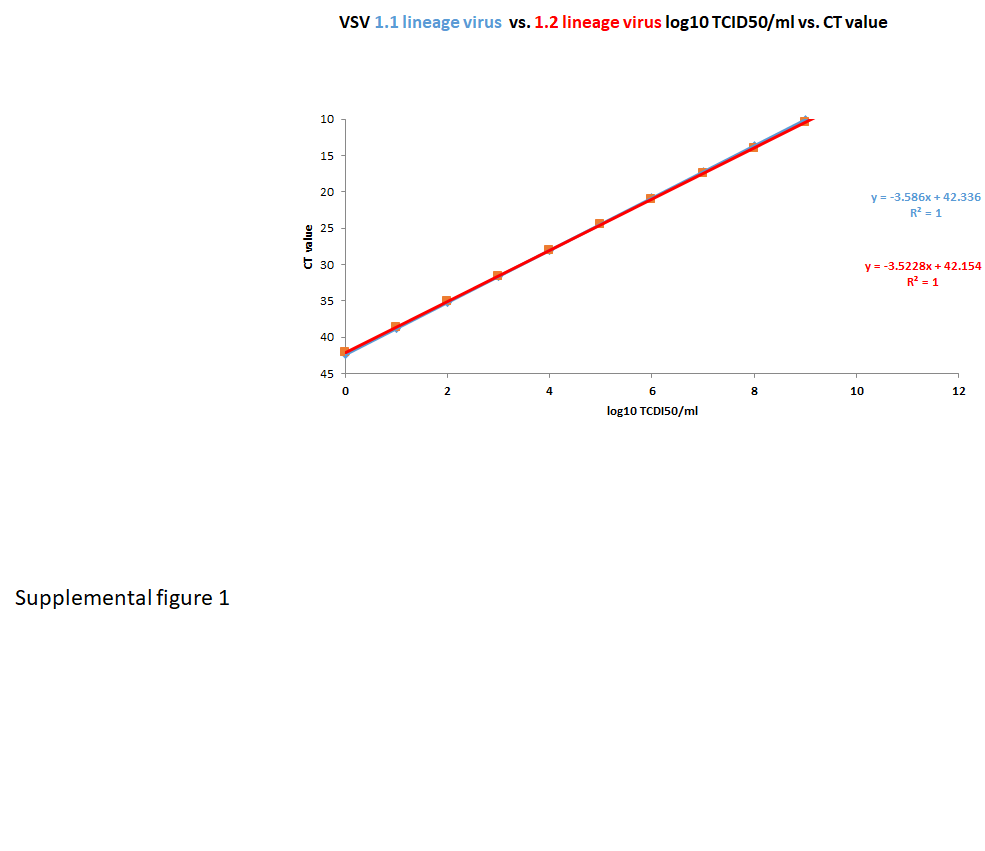

Supplement: FIGURE S1 — Equal sensitivity of rRT-PCR to detect VSV viruses 1.1 and 1.2. [file Image_1.tif]

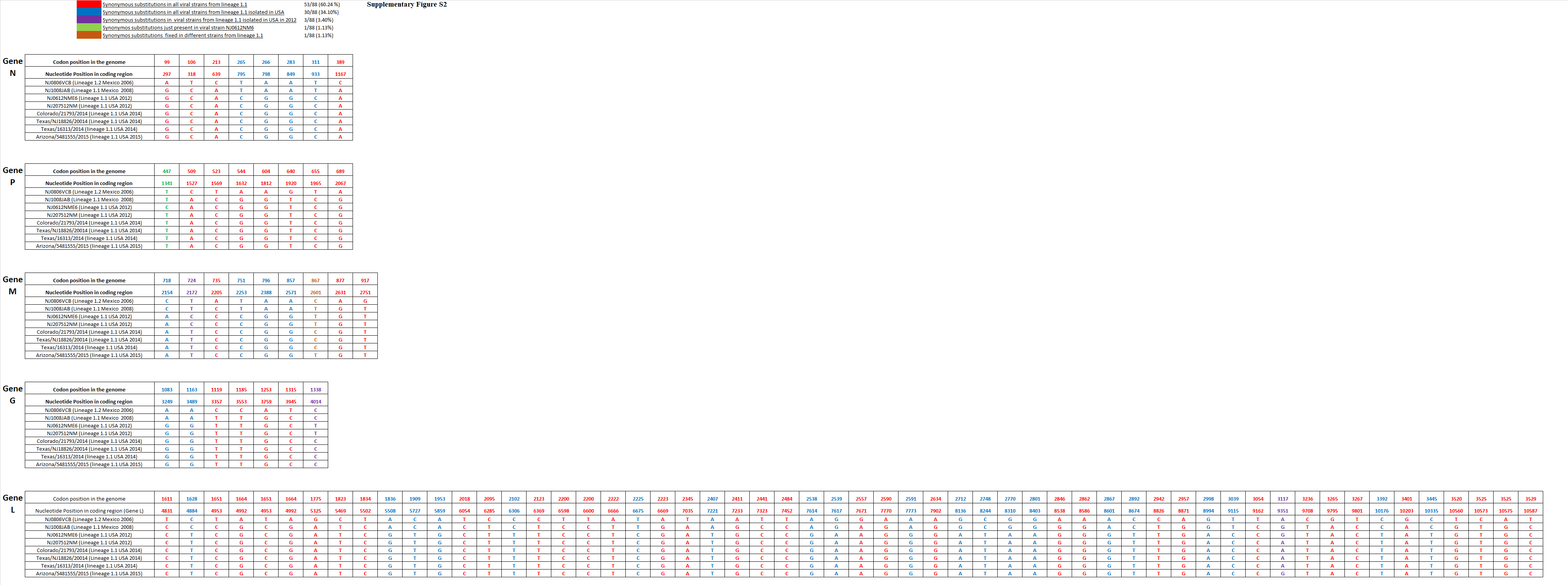

Supplement: FIGURE S2 — Synonymous substitution pattern on different genes associated with viral lineage 1.1. [file Image_2.tif]

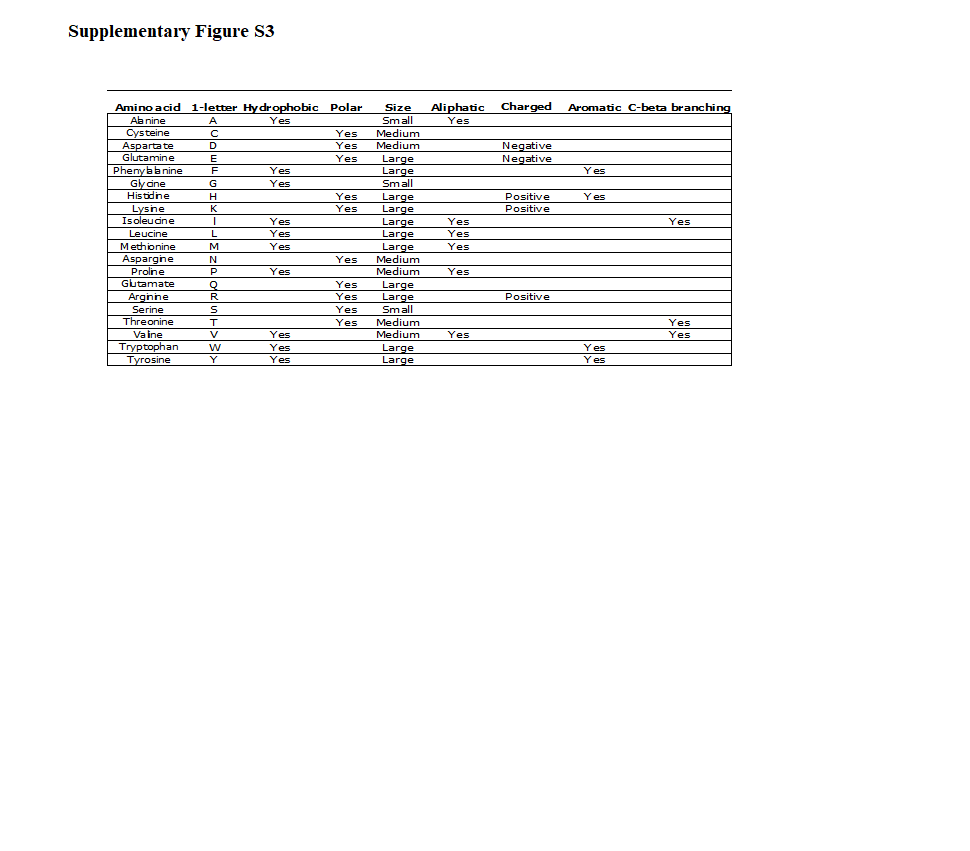

Supplement: FIGURE S3 — Amino acid chemical properties. [file Image_3.tif]
